# Less invasive methods of advanced hemodynamic monitoring: principles, devices, and their role in the perioperative hemodynamic optimization

**DOI:** 10.1186/2047-0525-2-19

**Published:** 2013-09-17

**Authors:** Christos Chamos, Liana Vele, Mark Hamilton, Maurizio Cecconi

**Affiliations:** 1 Senior clinical fellow in cardiac anaesthesia, St George’s Healthcare NHS Trust, London, UK; 2 Specialist registrar in anaesthesia, Croydon Health Services NHS Trust, London, UK; 3 Consultant and honorary senior lecturer in anaesthesia and intensive care medicine, St George’s Healthcare NHS Trust, London, UK

**Keywords:** Minimally invasive monitoring, Pulse pressure analysis, Lithium dilution, Transpulmonary thermodilution, Oesophageal doppler, Gas rebreathing, Transthoracic bioimpendance, Goal-directed therapy

## Abstract

The monitoring of the cardiac output (CO) and other hemodynamic parameters, traditionally performed with the thermodilution method via a pulmonary artery catheter (PAC), is now increasingly done with the aid of less invasive and much easier to use devices. When used within the context of a hemodynamic optimization protocol, they can positively influence the outcome in both surgical and non-surgical patient populations. While these monitoring tools have simplified the hemodynamic calculations, they are subject to limitations and can lead to erroneous results if not used properly. In this article we will review the commercially available minimally invasive CO monitoring devices, explore their technical characteristics and describe the limitations that should be taken into consideration when clinical decisions are made.

## Introduction

The need for the precise quantification of cardiac output (CO) in high-risk surgical patients, both in the operative room and the intensive care unit, is vital in modern medical practice. While up to 20 years ago CO had to be estimated from the PAC, nowadays new, less invasive techniques are available. When used together with perioperative protocols aiming at improving CO and oxygen delivery (DO2), their use is referred to as hemodynamic optimization or goal-directed therapy (GDT) [1].

Much has changed since the introduction of the pulmonary artery catheter (PAC) by Swan and Ganz in 1970 for the measurement of CO using the thermodilution method. Although in the context of moderate and high-risk surgery the beneficial effect of the PAC combined with goal directed therapy (GDT) has been established in a recent meta-analysis [2], the invasive nature of the insertion of the catheter and a considerable number of complications that follow its use (infection, arrhythmias, thrombosis, and pulmonary artery rupture) have led to a decline in its popularity, and prompted the scientific community to search for alternative methods that could substitute the PAC.

The term ‘minimally invasive cardiac monitoring’ encompasses all the methods and devices that calculate the cardiac output without the need of inserting a PAC, ranging from methods almost non-invasive to marginally less invasive than the PAC. These include the pulse pressure analysis, the transpulmonary thermodilution, the indicator dilution, the esophageal Doppler, the thoracic electrical bioimpedance, the carbon dioxide rebreathing, and the echocardiography. Since each one of these devices utilizes a different method of estimating the cardiac output, the clinician should be aware of their distinct features, their limitations but also the sources of potential error that stem for their use.

## Review

### Pulse pressure analysis

The pulse pressure analysis uses the arterial waveform, obtained either from an arterial catheter or a finger probe, in order to calculate the stroke volume (SV) and the systemic vascular resistance (SVR).

Its principles first described by Erlanger and Hooker in 1904 [3], pulse pressure analysis is based on the hypothesis that the SV is proportional to the arterial pulse pressure. However, a major drawback was the fact that the compliance of the aortic wall is non-linear rather than linear, being high at low distending pressures but decreasing more rapidly at higher pressures, preventing this way the overstretching of the vessel wall.

**Figure 1:**
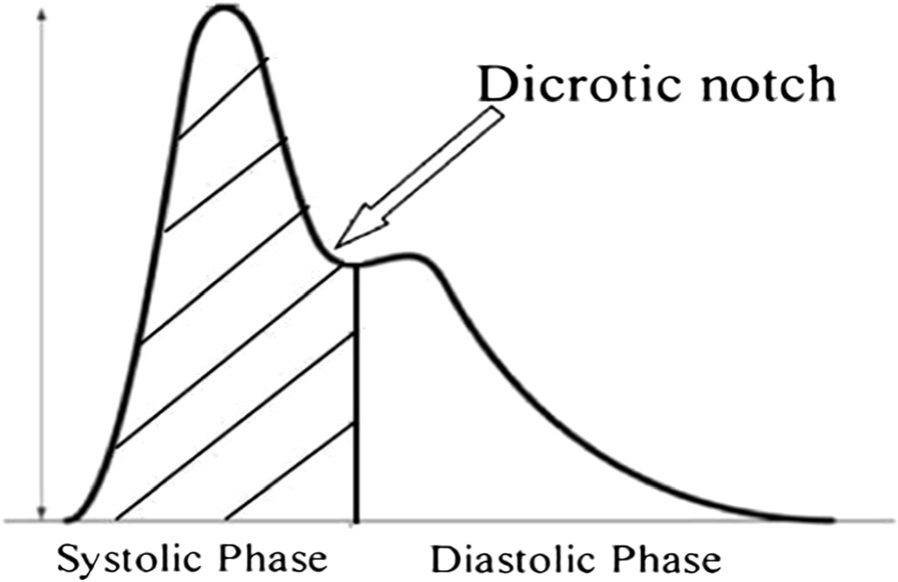
**The area under the curve (AUC) of the systolic phase is indicative of the SV.** The SV is the shaded area under the systolic portion of the arterial pulse waveform. It is calculated as=[area under systolic phase+ aortic compliance] x shape of pressure curve.

This and the fact that the compliance is also age-related, prevented any straightforward correlation of the pressure to the volume [4]. Only in 1983 and after the development of an algorithm by Wesseling et al. [5] to compensate for this non-linearity, did it become possible to calculate the SV by integrating the area under the curve of the systolic phase of the arterial waveform, and calculating the CO by simply multiplying the SV with the heart rate (HR) Figure 1.

One should always keep in mind that the pulse pressure method relies heavily on an optimal arterial pressure tracing, making an under- or over-damped arterial waveform a potential source of errors in calculation. Moreover, the pulse pressure systems necessitate an arterial wave that is purely reflective of the forward SV. As a consequence, situations in which the arterial wave is distorted either by artifact or a physiologic phenomenon (intra-aortic balloon counterpulsation, aortic regurgitation) will lead to inaccuracies [6]. Finally, the discrepancy in the compliance of the aorta compared to more peripheral parts of the arterial tree distorts the pattern of the arterial waveform and requires a careful interpretation of the measured values.

A variety of commercial systems that make use of the pulse pressure analysis method are available and they are divided in two groups, depending on the way that they are calibrated. Monitors that are ‘autocalibrated’ consist of the FloTrac/Vigileo, the Pulsioflex, the LidCO rapid, and the recently introduced Nexfin and esCCO monitors. On the other hand, there are devices that are externally calibrated: the PiCCOplus and the recently developed EV1000 use the transpulmonary thermodilution method, while the LidCO plus utilizes the lithium dilution technique for the same purpose. Moreover, apart from being useful tools in CO calculations, these monitors can also help predict the fluid responsiveness, as will be discussed later in this review article.

### Uncalibrated devices

Connected to a standard indwelling arterial catheter, the FloTrac sensor (Edwards Lifesciences, Irvine, CA, USA) uses an upgraded algorithm that derives the SV from the pulse pressure (PP) of the arterial waveform, after correcting for the compliance and the resistance of the arterial system.

A similar pulse pressure analysis method is used by the ProAQT sensor which is incorporated in the Pulsioflex monitor (Pulsion Medical Systems, Munich, Germany). Utilizing an existing peripheral arterial catheter and analyzing the arterial waveform 250 times per second, a start value for CO trend monitoring is determined after the patient’s characteristics are inserted to the system. To increase accuracy, a CO value measured by another method (for example by echocardiography) can be entered and thus the system can be externally calibrated.

As for the LidCO rapid system (LidCO Ltd, Cambridge, UK), it is based on the same algorithm as the LidCO plus monitor (which will be described later), but instead of thermodilution it relies on nomograms for the calculation of the CO.

One of the latest additions to the field of minimally invasive CO monitoring is the Nexfin monitor (BMEYE, Amsterdam, The Netherlands). Rather than a minimally invasive monitor, it is a completely non-invasive method of determining the patient’s hemodynamic parameters, as the need for an invasive arterial catheter is obviated. The monitor is connected to the patient by wrapping an inflatable cuff around the middle phalanx of the finger. The pulsating finger artery is ‘clamped’ to a constant volume by applying a varying counter pressure equivalent to the arterial pressure resulting in a pressure waveform. The finger arterial pressure is then reconstructed into brachial arterial pressure waveform using a transfer function and a level correction based on a vast clinical database. The resulting brachial pressure waveform serves as the basis for determining continuous CO. Real-time continuous CO and other hemodynamic parameters are derived by a novel pulse contour method (Nexfin CO-Trek®), which is based on the systolic pressure area and a physiological three-element Windkessel model individualized for each patient. Moreover, the presence of a co-oximeter enables the non-invasive calculation of hemoglobin, from which the oxygen delivery index (DO_2_I) is derived.

For completion purposes, we should also mention the latest entry in the pulse pressure analysis field, under the name of esCCO (Nihon Kohden, Tokyo, Japan). Also being a non-invasive monitor, it uses a new technology that derives the CO using the Pulse Wave Transit Time (PWTT), which is obtained by the pulse oximetry and the ECG signals of each cardiac cycle. The rationale behind this is that animal studies have shown a strong correlation between the SV and the PWTT. The only up-to-date validation study has demonstrated a clinically unacceptable performance of the device when compared to the CO values obtained with transthoracic echocardiography [7].

The accuracy of the pulse pressure analysis method in estimating CO has been extensively investigated against CO calculations by the thermodilution method using the PAC. This holds true particularly for the FloTrac/Vigileo monitoring. In one of the most recent meta-analyses, Mayer et al. [8] showed improved correlation between the Flotrac/Vigileo and the thermodilution method when the new generation software was used, as opposed to the initially poor correlation with previous generation softwares. Also, the SVV calculated with this method showed good performance in predicting fluid responsiveness in septic shock patients [9]. On the other hand, it appears that the CO calculated by the FloTrac/Vigileo has a less accurate correlation with the one derived by the PAC when changes are induced by norepinephrine administration [10], and also tends to be overestimated compared to a continuous cardiac output calculation with a PAC when used in off-pump coronary artery bypass surgery (CABG) [11]. A discrepancy in measurements also appears when the FloTrac/Vigileo is used to calculate hemodynamic parameters in open aortic abdominal aneurysm (AAA) repair [12] compared with echocardiography derived measurements.

Given its straightforward setup and use, it seems that the FloTrac/Vigileo is a useful tool correlating well with the gold standard of the thermodilution method with the PAC, in patients with a regular rhythm and stable respiratory pattern and mechanics, hospitalized in an environment with less abrupt hemodynamic changes as is the intensive therapy unit (ITU). While awaiting for further studies to assess this relationship, a more cautious approach should be adopted when it is used to assess the CO in patients that rely on substantial inotropic or vasopressor support or in environments and settings with rapid dynamic changes, as in the operating room (OR).

Despite its relatively short presence in the market, the non-invasive Nexfin monitor has also been validated against the traditional methods of CO calculation, yielding positive results so far. Two different research groups demonstrated a reasonable correlation of CO values obtained by the Nexfin when they were compared with those calculated by transpulmonary thermodilution, both in the setting of cardiac surgery [13,14]. The same good agreement appears when the Nexfin derived CO values are compared to those measured by Doppler echocardiography, either from the transthoracic [15] or the esophageal route [16]. Adding to these studies the lack of invasiveness and the ease of use, the Nexfin monitor appears to be an alternative method of calculating hemodynamics where the traditional invasive approach is difficult or undesirable.

### Calibrated devices

**Figure 2:**
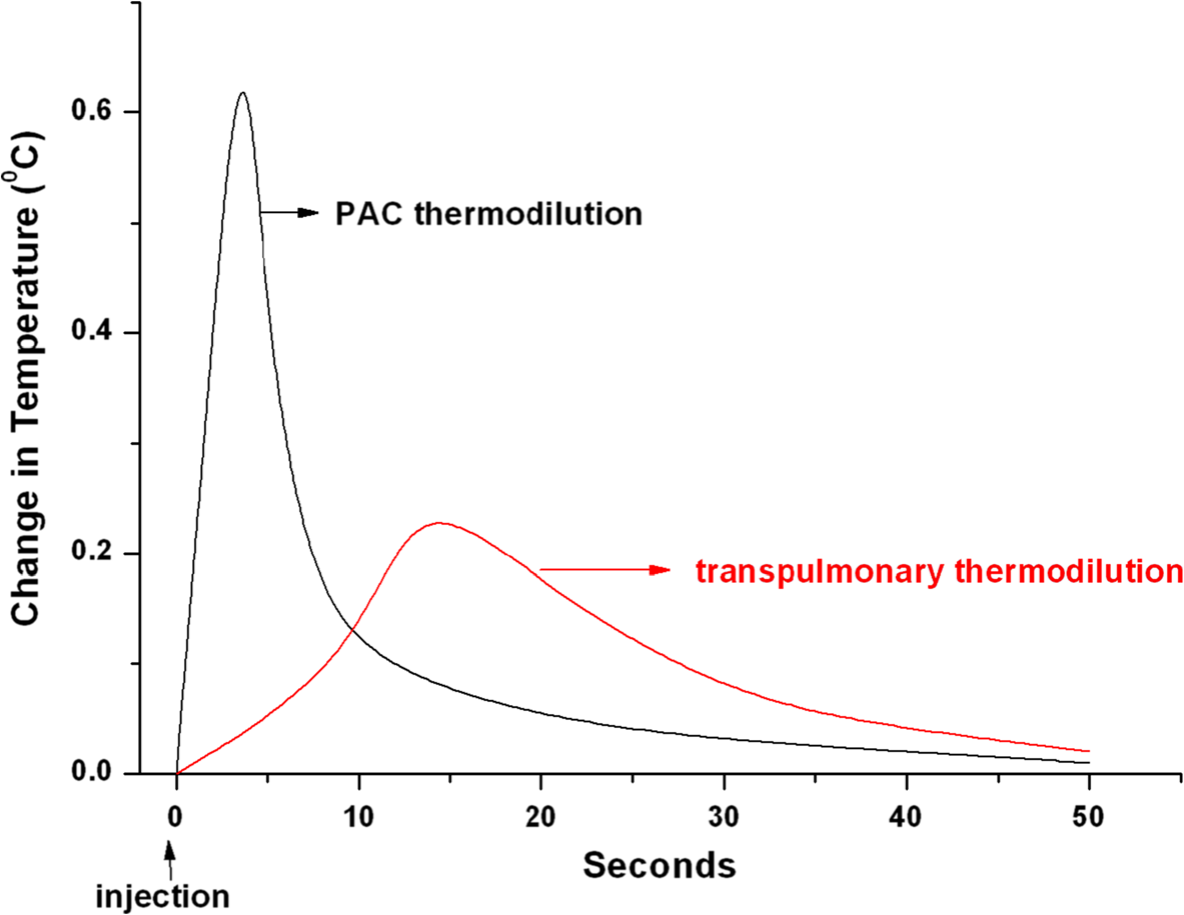
**The transpulmonary thermodilution curve, with the characteristic delay in the peak temperature change compared to the PAC thermodilution.** Based on the same principle as the PAC thermodilution, the transpulmonary method obviates the need for a PAC.

The PiCCOplus monitor (Pulsion Medical Systems, Munich, Germany), which is based on the pulse pressure analysis to provide continuous real-time assessment of the CO, uses the transpulmonary thermodilution for intermittent calibration, being until recently the only device to incorporate this method in a commercial application. This method is based on the same principle as the traditional thermodilution but spares the need for a PAC insertion. The technique starts with the injection of a cold injectate in the superior vena cava (SVC) via a central venous catheter (CVC) which mixes with the ambient blood and travels through the right heart, the pulmonary vasculature and the left heart before reaching the aorta. A thermistor in the aorta or a major arterial branch measures the blood temperature and a thermodilution curve of temperature over time, similar to that from the PAC-based method but having a delayed peak temperature change, is plotted. The CO is measured by the thermodilution equation:

$$\mathrm{CO}=\frac{\left(T_{B}-T_{I}\right)\times K}{\int_{0}^{\infty }{\mathrm{\Delta Τ}}_{B}\left(t\right)\mathrm{dt}}$$

here T_B_=blood temperature, T_I_=injectate temperature, and K=−computation constant (Figure 2).

The PiCCOplus monitor also provides information on variables as the global end-diastolic volume (GEDV) [17], which is an estimate of the preload much more reliable than the CVP, the intrathoracic blood volume (ITBV), the extravascular lung water (EVLW), and the pulmonary vascular permeability index (PVPI). The combination of the last two variables could potentially help in diagnosing pulmonary edema and distinguishing between the hydrostatic and inflammatory forms of the condition [18]. This comes at the cost of needing a CVC for injection of the cold indicator and a thermistor-tipped catheter in a central artery (femoral, axillary, or brachial), this way being a more invasive method than the FloTrac/Vigileo and the LidCO monitors.

Since a pulse contour method is used for continous CO display, the same limitations as in the FloTrac/Vigileo monitor apply for the PiCCOplus (IABP counterpulsation, significant aortic regurgitation, arrhythmias). Sources of potential errors that are specific for the transpulmonary thermodilution come from the fact that the PiCCOplus estimates the CO of the left heart whereas the PAC thermodilution that of the right side. These should be identical in ideal conditions, however a discrepancy is encountered in the presence of intracardiac and intrapulmonary shunts. Moreover, conditions related to indicator loss in the tissues (that is, in the presence of pulmonary edema) or recirculation will lead to a discrepancy of the calculated CO compared to the one derived by the PAC thermodilution, although these two phenomena may actually cancel each other [19].

Regarding the reliability of the method, it appears that there is good correlation of the CO measurements obtained with the PiCCOplus compared to other methods. Two studies performed on pediatric animal models by the same scientific team showed a significant correlation when the transpulmonary thermodilution derived values were juxtaposed to those obtained with an ultrasound probe around the main pulmonary artery, both in normal cardiac anatomy and in the presence of a left-to-right shunt [20,21].

An older study confirms the good performance of the transpulmonary thermodilution even during substantial variations in vascular tone and hemodynamics [22]. Taking these under consideration and calibrating the device according to the manufacturer’s advice, it is reasonable to consider the PiCCOplus as a reliable, less invasive substitute method to the PAC.

The latest entry in the area of minimally invasive CO monitoring using the transpulmonary thermodilution comes from Edwards Lifesciences, which developed the EV1000/VolumeView monitor (Edwards Lifesciences, Irvine, CA, USA). Needing, as the PiCCOplus does, a specific to the set central arterial catheter and using a patented for the company algorithm, it also displays volumetric parameters as the EVLW and GEDV but also a new variable named the global ejection fraction (GEF). A recent clinical validation study proved the interchangeability of this new method to the PiCCOplus monitor, with VolumeView scoring better in the calculation of GEDV [23].

The last of the calibrated CO monitors is the LidCOplus (LidCO Ltd, Cambridge, UK) that utilizes the lithium dilution technique. Based on the pulse power rather than the pulse pressure analysis via the PulseCO algorithm, which does not rely on the arterial waveform morphology, and needing only a peripheral arterial catheter, the LidCOplus technology uses the lithium dilution to intermittently calibrate the system. Specifically, boluses of 0.5-2 mL of lithium chloride are each time injected through a peripheral or central line and the lithium concentration is measured through aspiration of blood from the arterial catheter, using a Li^+^-sensitive electrode attached to the catheter that generates a voltage [24]. Since the electrode has a low sensitivity for distinguishing lithium from sodium, a correction factor is applied for sodium plasma levels and a baseline voltage is determined that helps differentiate the concentration of the two cations. Once the lithium dilution curve is obtained, the CO is calculated using the Stewart-Hamilton equation, again with a correction for packed cell volume. The main advantage of lithium as an indicator is that it does not naturally occur in plasma and therefore can generate a high signal-to-noise ratio when used with a sensitive electrode, followed by a rapid redistribution time and an insignificant first-pass loss from the circulation [25].

Apart from the expected lack of accuracy of the LidCOplus technology in a patient already on lithium treatment, the other interference that should be taken into account is the use of bolus doses of muscle relaxant drugs in the operative and intensive care setting. High peak doses of these drugs can cross-react with the lithium sensor as they incorporate a positively charged quaternary ammonia ion that can be detected by the sensor and thus lead to an overestimation of the CO. Muscle relaxant agents which are not compatible with the LidCOplus monitor are atracurium and rocuronium, whereas suxamethonium, vecuronium, and pancuronium can be used provided that a time interval of 15–30 minutes elapses between their bolus administration and the LidCOplus calibration.

The consistency of the CO measurement with the LidCOplus technology compared to the traditionally established thermodilution method with the PAC, has been investigated in a number of validation studies. In one of the most recent studies by Mora et al. [26], the lithium dilution method showed good correlation and marginal bias (0.28 L/min) with the thermodilution method in patients with impaired left ventricular function after cardiac surgery. This good correlation was corroborated by Costa et al. [27] when they validated the LidCOplus against intermittent thermodilution measurements in patients with hyperdynamic conditions. On the other hand, the uncalibrated pulse power analysis using the Pulse CO algorithm performed less well when used in comparison to the PAC based thermodilution in patients undergoing CABG [28] or when used in very dynamic conditions such as the clamping and unclamping of the aorta in AAA surgery [29]. Concluding, the LidCOplus technology appears to be a reliable substitute to the more invasive thermodilution method via the PAC, provided that the system is calibrated in regular intervals when an absolute value rather than a simple trend of the CO is required.

### Esophageal Doppler monitor

The Esophageal Doppler (ED) monitor, which is based on the Doppler effect in order to measure the velocity of blood flow, was first introduced in the 1970s as a non-invasive means to measure CO. The velocity is calculated from the following equation:

$$V\left(cf_{d}\right)/\left(2f_{0}\cos\theta \right)$$

where v is the blood flow velocity, c is the speed of sound in tissue, f_d_ is the frequency shift, f_0_ is the frequency of the emitted ultrasound, and θ is the angle between the ultrasound beam and the direction of the blood flow. If the flow of blood in the descending aorta is known, this figure can be used to estimate the stroke volume and hence the cardiac output. The esophageal Doppler measures the velocity of blood in the descending aorta in centimeters per second (cm/s). In order to convert this figure into blood flow in milliliters per second (mL/s), the diameter of the aorta needs to be known. This is derived from published nomograms based on age, sex, weight, and height (Deltex, West Sussex, England) or through direct ultrasound measurement (Arrow’s HemoSonic® Reading, PA, USA). The ED also has the ability to measure the corrected flow time (FT_c_) as a measure of cardiac preload. The FT_c_ is the duration of flow during systole corrected for a heart rate of 60 beats per minute. It is unclear as to whether the FTc or SV should be used to guide fluid therapy, but it appears that the ability to respond to a fluid challenge is best determined by FTc. Several studies have compared FT_c_ with other indices such as pulmonary artery occlusion pressure and have found good agreement between the two [30-32].

There are some limitations to the usage of ED. First, the ED only measures descending aortic blood flow, which may not always be constant due aortic pathology or compression or due to abnormal upper to lower body blood flow distribution. Also, the aortic cross-sectional area is not constant, due to changes in pulse pressure, vascular tone, aortic compliance, volume status, or catecholamine use. Due to the fact that the radius of the aorta is squared in the final CO equation, even small changes in aortic area can significantly affect CO determinations [33]. Moreover, the probe position is critical to obtain accurate measurement for both blood flow measurement and aortic cross-sectional measurement. The Doppler beam must be within 20° of axial flow to obtain a good measure of aortic blood flow. Even small misalignments of the ultrasound beam with blood flow will lead to underestimation of flow when using the Doppler equation [34,35]. Finally, the equation assumes that the flow is laminar and any turbulent flow in the aorta will reduce measurement accuracy.

There have been studies that have compared ED measurements of CO with PAC-derived thermodilution CO. Dark and Singer performed a literature review regarding the validity of ED monitoring as a measure of CO in critically ill adults, concluding that the ED monitor has high validity in tracking changes in CO [36]. In addition, other studies have shown that using ED to guide fluid administration improved patients’ management. Sinclair et al. conducted a randomized controlled trial of patients undergoing femoral fracture repair. Patients were randomized to either routine care or ED-guided fluid loading. The patients in the ED group had a shorter hospital stay [37]. A similar trial conducted by Venn et al. showed that the ED-monitored patients had less intraoperative hypotension and were considered fit for discharge earlier [38]. Noblett et al. [39] performed a double-blind randomized control trial of Doppler-guided fluid therapy *versus* anesthetist-directed fluid therapy in 108 patients undergoing elective colorectal resection. They found that despite both groups of patients receiving the same volume of intraoperative fluids, the group managed with the ED had a shorter hospital stay and lower morbidity rates than the control group. Interestingly, patients in the intervention group also had lower levels of interleukin-6, which suggested an attenuated inflammatory response to surgery, perhaps due to the improved organ perfusion. In a study by Wakeling et al. patients recovered gut function significantly faster and suffered significantly less gastrointestinal and overall morbidity [40]. These overwhelming data in support of the ED led the National Institute for Health and Clinical Excellence (NICE) to release guidelines in 2011 advocating the use of this technology [41].

A completely non-invasive Doppler technology, the USCOM (Ultrasound CO monitor, USCOM, Sydney, Australia), is also available which uses Doppler technology to measure CO from a suprasternal Doppler probe. This technology has been studied in a few patient population groups (mostly stable ICU patients) and has shown reasonable correlation with PAC [42,43].

### Thoracic electrical bioimpedance

Variations in the electrical impedance of the thorax to an alternating current which occur synchronously with the cardiac cycle were observed nearly 40 years ago. The first use of this phenomenon to measure SV and CO was described by Nyboer [44], and Kubicek and colleagues [45] introduced the technique into clinical practice in 1966.

Electrical bioimpedance involves the analysis of intrabeat variations in transthoracic voltage in response to the applied high frequency transthoracic current. Two commercial devices based on electrical bioimpedance use electrodes attached to an endotracheal tube (ECOM, Conmed Corp, Utica, NY, USA) or the skin (BioZ, Cardio-Dynamics, San Diego, CA, USA). Pulsatile changes in thoracic blood volume result in changes in electrical impedance. The rate of change of impedance during systole is measured and an estimate of the SV and the CO is derived from a mathematical equation.

Sources of potential inaccuracies include motion artifacts, electrical interference, cardiac arrhythmias, heart and lung pathologies (as chest deformities, pulmonary edema, pleural and pericardial effusions, intracardiac shunts), and foreign bodies (as chest tubes). Clinical trials of TEB have been shown to be reliable in young healthy volunteers, but in septic or surgical patients, the results have been inconsistent [46-48].

### Bioreactance

Recently, bioreactance (NICOM, Cheetah Medical Ltd, Maidenhead, Berkshire, UK), a modification of thoracic bioimpedance, has been introduced. Bioreactance refers to the electrical resistance, capacitive and inductive properties of blood and biological tissue that induce phase shifts between an applied electrical current and the resulting voltage signal. In contrast to bioimpedance, the bioreactance technique analyzes the frequency spectra variations of the delivered oscillating current. When blood flows out of the heart, phase shifts occur if alternating currents are applied across the patient’s chest. Such phase shifts are conceptually similar to a frequency modulation as used in radio transmission. The phase shifts are measured continuously and have been shown to relate almost linearly to blood flow in the aorta. This results in less interfering from the electrical noise, patient movement, respiratory effort, lead placement, and body mass index.

In addition to cardiac output, mean bioreactance measurements are indicative of the total thoracic fluid content (TFC). TFC is affected by both intravascular and extravascular fluid in the chest cavity and although it does not correlate with pulmonary artery wedge pressure (PAWP), its changes are very reliable indicators of the changes in intravascular or extravascular fluid volume [49]. In a study of patients undergoing cardiac surgery, the bioreactance did well initially in determining CO when compared with PAC, however, during the immediate postoperative period, the correlation was not as robust [50].

It appears that the new generation of devices might be better than the first generation TEB machines, although there are still limitations regarding their accuracy in measuring CO during dynamic conditions.

### Gas rebreathing

The partial carbon dioxide (CO2) rebreathing technique uses the Fick principle applied to the CO2 in order to estimate CO non-invasively. The NICO monitor (Novametrix Medical Systems, Inc., Wallingford, CT, USA) implements intermittent partial rebreathing through a specific disposable rebreathing loop. The monitor consists of a CO2 sensor, a disposable airflow sensor and a pulse oximeter. CO2 production (VCO2) is calculated from minute ventilation and its CO2 content, whereas the arterial CO2 content (CacO2) is estimated from end-tidal CO2, with adjustments for the slope of the CO2 dissociation curve and the degree of dead space ventilation. The partial rebreathing reduces CO2 elimination and increases end-tidal CO2. Measurements under normal and rebreathing conditions allow one to omit the venous CO2 content (CvCO2) measurement in the Fick equation, because CvCO2 does not change during this brief period of rebreathing [51].

$$\mathrm{CO}=\mathrm{VCO}2/\left(\mathrm{CvCO}2‒\mathrm{CaCO}2\right)$$

While the technique is easy to use, the correlation between the NICO and standard thermodilution has been shown to be adversely affected in spontaneously breathing patients, which limits the number of suitable candidates [52,53]. Partial rebreathing has been shown to be more accurate in less critically ill patients with normal alveolar gas exchange when compared with PAC thermodilution [54,55]. Severe chest trauma, significant intrapulmonary shuntdead-space ventilation, low minute ventilation, and high CO may all reduce accuracy [56]. There have been no reports on the device when used in hemodynamically unstable patients [57]. Moreover, the partial-rebreathing technique only measures the CO and does not provide information on the intravascular volume status or fluid responsiveness. For these reasons, partial gas rebreathing is limited in its clinical applicability.

### Transesophageal echocardiography

Widely used in the cardiac operative and postoperative setting to demonstrate cardiac anatomy and identify pathology, the transesophageal echocardiography (TOE) has been impractical in assisting with continuous CO measurements outside the operative theater because of the size of the probe and the limitations that come with it. However, the recent development of a miniaturized 5 mm TOE probe (ClariTEE Probe, ImaCor Inc., Garden City, NY, USA) that can stay indwelled for up to 72 hours, has enabled hemodynamic management of the critically ill patients [58]. Although not a CO monitoring *per se*, the ClariTEE uses a monoplane transducer that enables the operator to acquire basic views of the heart (ascending aortic short-axis, four-chamber, and transgastric short-axis) that, combined with a software tool, offer continuous calculations of the ventricular size and systolic performance. Moreover, echocardiography derived measurements of the stroke volume can help in the assessment of fluid responsiveness [59]. Validation studies are pending.

### Predicting fluid responsiveness

The assessment of intravascular volume status of an individual has been traditionally performed with static parameters, mainly filling pressures such as CVP and PAWP, which reflect the preload of the right and the left ventricles, respectively. It is now acknowledged that these values correlate poorly with the intravascular volume and the response to a fluid bolus [60], hence there has been a shift to more dynamic indices such as the stroke volume variation (SVV) and the pulse pressure variation (PPV).

**Figure 3:**
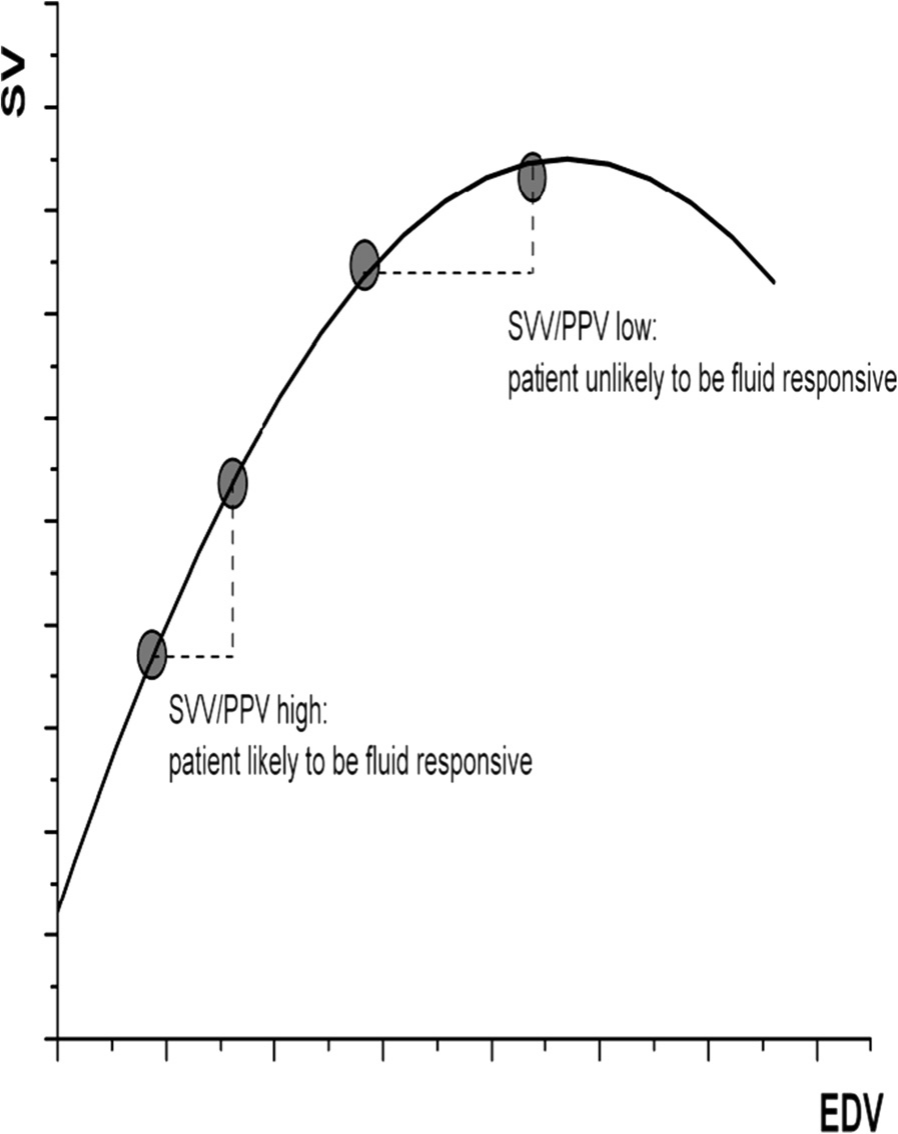
**Correlation of fluid responsiveness with SVV/PPV variables.** As a general rule, the higher the SVV/PPV values, the more fluid responsive the patient will be. The correlation can only be applied to mechanically ventilated patients.

All the devices that use the pulse pressure analysis method for CO monitoring, also calculate those two parameters. Based on the effect of the cyclic variation of intrathoracic pressures with respiration on the preload of the left ventricle and using appropriate algorithms, the beat to beat variation of the SV and the pulse pressure (PP) is calculated. Specifically, positive pressure ventilation induces cyclic changes in vena cava blood flow, pulmonary artery flow, and aortic blood flow. During inspiration, vena cava blood flow decreases (venous return decreases) and, according to the Frank-Starling relationship, pulmonary artery flow decreases. Approximately three beats later this decrease in pulmonary artery flow is transmitted to the left ventricle inducing a decrease in SV and aortic PP. As a general rule, values of SVV >10% and PPV >13% are indicative of patients that will probably respond to the administration of fluids, while lower values indicate the opposite. The basic limitation is that the above calculations can only be applied to mechanically ventilated patients, as the correlation in spontaneously breathing individuals is weaker. Moreover, for optimal results the tidal volume (TV) should be >8 mL/Kg of body weight, the patient should be in sinus rhythm, and the chest must be closed [61] (Figure 3).

More recently, a new index called the Pleth Variability Index (PVI) may help with the prediction of fluid responsiveness. The PVI is continuously calculated by the new non-invasive Masimo monitor (Masimo Corporation, Irvine, CA, USA), a device that resembles the traditional pulse oximeter. The monitor measures first the change during a respiratory cycle of the Perfusion Index (PI), which is the ratio of non-pulsatile to pulsatile blood flow through the peripheral capillary bed, and then calculates the PVI from the following formula:

$$\mathrm{PVI}=\left[\left(\mathrm{PImax}‒\mathrm{PImin}\right)/\mathrm{PImax}\right]x100$$

The greater the PVI value is, the more likely the patient will respond to fluid administration [62]. As is the case with SVV and PVV, one should always bear in mind the same limitations before taking clinical decisions regarding the hemodynamic optimization of patients (Table 1).

**Table 1:** Overview and classification of minimally invasive CO monitors

**Modality**	**Available devices**	**Requirements**	**Additional values**
Pulse pressure analysis			
-Uncalibrated devices	Flo/Trac-Vigileo	Standard arterial line	GEDV, EVLW
	Pulsioflex	Standard arterial line	
	LidCO rapid	Standard arterial line	
-Calibrated devices	LidCO plus	Standard arterial line	
	PiCCOplus	Standard arterial line	
		Central venous line	
	Volumeview	Standard arterial line	
		Central venous line	GEDV, EVLW, GEF
Doppler			
-Transesophageal	CardioQ/CardioQ-OCM	Transesophageal probe	FTc
-Transthoracic	USCOM	Transthoracic probe	
Echocardiography	ClariTEE	Miniaturized transesophageal probe	
Gas rebreathing	NICO	Disposable rebreathing circuit	
Bioimpedance/ Bioreactance			
-Thoracic electrical bioimpedance			
- Bioreactance	BioZ	Specific electrodes	
	NICOM	Specific electrodes	
Non-invasive monitors			
	Nexfin	Finger probe	Hb
	Masimo	Specific pulse oximeter	PVI
	esCCO	ECG/ specific pulse oximeter	PWTT

### Goal-directed therapy

All the above methods of quantifying the CO have the potential to reduce the morbidity and mortality of the patients if they are integrated into appropriate protocols that guide the therapeutic interventions. The use of CO measurements to guide fluid administration and inotropic therapy, optimizing this way the tissue perfusion and cellular oxygenation has been given the broad term goal-directed therapy (GDT). Its beneficial effects demonstrated as early as the late 1970s by the seminal work of Shoemaker and his team [63,64], it is now accepted that GDT reduces complications and improves outcome rates in both surgical [2,65,66] and non-surgical populations [67]. With increasing availability of a variety of minimally invasive CO monitors, one should expect the GDT to be widely implemented for the hemodynamic optimization of various patients’ groups, especially when questions regarding the cost-effectiveness of the method are adequately addressed.

## Conclusions

A whole array of minimally invasive devices and monitors that can reliably calculate trends in the CO without the need for a PAC are now available to the clinicians. Needing only peripheral or central arterial catheters or only a finger probe, as is the case in the Nexfin and Masimo monitors, they can give reliable measurements of CO and of dynamic indices such as the SVV and the PPV, provided that their limitations are taken into account.

Additional tools in the area of minimal invasiveness are the OD, with its use strongly advocated by recent NICE guidelines, and the less widely used gas rebreathing and thoracic bioimpedance methods. Last, the introduction of a miniaturized TOE probe may revolutionize the management of ITU patients, providing real-time information on cardiac anatomy and function.

The integration of these devices into therapeutic protocols enables the clinician to apply GDT and guide the inotropic support and fluid administration in a rational way, reducing this way the mortality and morbidity of the patients.

## Abbreviations

AAA: Abdominal aortic aneurysm; BP: Blood pressure; CABG: Coronary artery bypass grafting; CI: Cardiac Index; CO: Cardiac output; CVC: Central venous catheter; CVP: Central venous pressure; DO2: Oxygen delivery; DO2I: Oxygen delivery index; ED: Esophageal Doppler; EPCI: Ejection phase contractility index; EVLW: Extravascular lung water; FTC: Corrected flow time; GDT: Goal-directed therapy; GEDV: Global end-diastolic volume; GEF: Global ejection fraction; HR: Heart rate; IABP: Intra-aortic Balloon Pump; ITBV: Intrathoracic blood volume; ITU: Intensive Therapy Unit; NICE: National Institute for Health and Clinical Ecxellence; OR: Operating room; PAC: Pulmonary artery catheter; PP: Pulse pressure; PAWP: Pulmonary artery wedge pressure; PWTT: Pulse wave transition time; PI: Perfusion index; PPV: Pulse pressure variation; PVI: Pleth Variability Index; PVPI: Pulmonary vascular permeability index; SV: Stroke volume; SVC: Superior vena cava; TEB: Thoracic electrical bioimpedance; TOE: Transesophageal echocardiography; TV: Tidal volume; VEPT: Volume of electrically participating tissues; VET: Ventricular ejection time.

## Competing interests

CC and LV declare no competing interests. MH has received lecture fees and expenses from Deltex Medical, Edwards Lifescienses, LidCO. MC has received honoraria, travel expenses, or unrestricted educational grants from LiDCO, Edwards Lifesciences, Deltex, Cheetah, Masimo, and Bmeye; he is part of the medical advisory board of Applied Physiology.

## Authors’ contributions

CC has drafted the manuscript apart from the sections for the esophageal Doppler, the thoracic electrical bioimpedance, the bioreactance, and the gas rebreathing. LV has written the sections for the esophageal Doppler, the thoracic electrical bioimpedance, the bioreactance, and the gas rebreathing. MH has critically reviewed the manuscript. MC has conceived and critically reviewed the manuscript. All authors have read and approved the final version of the manuscript.
